# How to choose a preceptor: aspects to consider based on a grounded theory study

**DOI:** 10.1186/s12912-023-01240-w

**Published:** 2023-03-31

**Authors:** Guida Amaral, Amélia Simões Figueiredo

**Affiliations:** 1 Universidade Católica Portuguesa, Institute of Health Sciences, Instituto Politécnico de Setúbal, ESS, NURSE’IN-UIESI, Lisbon, Portugal; 2 Universidade Católica Portuguesa, Institute of Health Sciences, Centre for Interdisciplinary Research in Health, Lisbon, Portugal

**Keywords:** Nursing education, Preceptorship, Professional competence, Grounded theory, Nursing research

## Abstract

**Background:**

Nurses in clinical practice settings share responsibility with academy teachers in the education of nursing students in clinical teaching. This dynamic is essential for the students’ learning and their skill development. During preceptorship, the nurse has to mobilize the available resources to act competently and effectively. The purpose of this article is to identify the aspects that should be considered when selecting a nurse preceptor.

**Methods:**

A qualitative study was carried out based on Grounded Theory using Strauss and Corbin’s version. The study took place in a medicine ward and a surgery ward of a hospital in the Lisbon metropolitan area. The authors conducted fourteen semi-structured interviews with nurses who were at different stages of Benner’s Professional Development Model and with different outlooks on nursing preceptorship. The initial script was reorganized after the pilot test. It was also adjusted after the first exploratory interviews and after the in-depth interviews in order to reflect the interviewees’ work experience and outlooks on preceptorship. The interviews had an average duration of 45 min and were audio recorded. Theoretical sampling was conducted considering the needs identified in data coding until we reached theoretical saturation. Data analysis began after the first interview, first by open coding, followed by axial and selective coding, always in a logic of constant comparison with theory. Ethical principles, scientific integrity and methodological rigour were ensured throughout the study.

**Results:**

Two categories emerged that were supported by all nurses: skills and individual characteristics. The former includes communication skills, relational skills, reflective skills, technical-scientific skills, and emotional skills, while the latter includes perceptiveness, responsibility, motivation, and professional initiative.

**Conclusion:**

Nurses value both the individual characteristics they possess and the skills they have developed throughout their career. The recognition of the aspects found in this study for the selection of nurse preceptors allows for an informed and reasoned decision making, with a view to the success of the preceptorship and to the improvement of the quality of nursing education.

## Background

This article comes within the scope of a doctoral program in nursing education, whose general objective is to understand the process of skill development of the nurse preceptor during the practical component of the nursing degree, calledclinical teaching. [1]. The main objective of clinical teaching is for the students to acquire and develop knowledge, attitudes, and skills essential to the nursing professional practice [1].

Since nurses in clinical practice contexts collaborate with the professors of educational institutions during clinical teaching, they share the responsibility of training nursing students [2].

Furthermore, the dynamic that is established between the nurse preceptor and the student is fundamental for the student’s learning, acquisition and development of skills [3] which must be consistent with the general care nurse competency profile defined by the Order of Portuguese Nurses.

Mikkonen et al. [4] value the role of nurse preceptors in the training of students, especially in the development of their knowledge and skills in clinical teaching and which are the foundation of their professional development. Ignacio and Chen [5] add that one of the attributes of the preceptor is the ability to facilitate the cognitive integration of knowledge and of skills that are transferable to the clinical practice.

The responsibilities, attitudes, roles, abilities, skills, and characteristics of the nurse preceptor have been pointed out by different authors in international articles: Pearson and Hensley [6], Martínez-Linares et al. [7], Loughran and Koharchick [8], and Borrallo-Riego et al. [9].

In a systematic literature review carried out by Amaral and Figueiredo [10] whose objective was to identify the nurse preceptor skills in clinical teaching, six categories were identified: relational skills, analytical skills, reflective skills, pedagogical skills, learning skills and professional skills. Evidence points to relational skills as predictors of pedagogical skills. These are harmoniously anchored to the analytical and reflective skills that mediate the acquisition of learning skills and support professional skills in the preceptor’s performance [10].

Considering Le Boterf’s [11] concept of skills, for the nurse preceptor to be competent, they must know how to act in a responsible, effective and established manner in the face of a situation in a professional context. To act competently, the nurse preceptor must know how to select, mobilize, integrate, and transfer available resources. The author adds that the combination of the quality of both personal and environmental resources may influence the quality of the skills in action [11].

Regarding the socio-professional development of nurses, Benner [12] characterizes the acquisition of clinical skills in five stages: beginner, advanced beginner, competent, proficient, and expert. These stages consider the work context experience and reflect the evolution of the nurse’s performance, which emerges from the mobilization of life experience and the evolution of how the level of demand of the situations is understood.

It is important to clarify the aspects that facilitate the selection of the nurse preceptor not only because this role has a strong impact in the learning and development of the nursing student, but also because the nurse preceptor must effectively mobilise the desirable preceptor skills described in literature.

Therefore, the authors conducted an integrative review with the goal of describing the current evidence available on the development of the nurse preceptor’s skills. The four articles selected reveal that the peer support relationship and the identification of skill development needs are fundamental to create a flexible and creative curriculum. However, this integrative review also reveals lack of currently available evidence on the development of the nurse preceptor’s skills, which presented us with the need to further investigate this topic [1, 13].

This article purports to present one of the specific objectives of the research previously mentioned, whose general objective is to understand the process of skill development of the nurse preceptor in clinical teaching.

## Methods

### Purpose

The aim of this article is to present the aspects that should be considered when selecting a nurse preceptor, which we identified during the main research. Design.

In order to understand the process of skill development of the nurse preceptor in clinical teaching, we used a qualitative approach based on the grounded theory version of Strauss and Corbin (2014).

We chose this approach because it makes it possible to understand from the interviewee’s point of view the meaning of the phenomenon, the understanding of the experience, feelings, interactions and how meaning is extracted [14]. Through this approach, we were able to analyze in depth the practices and behaviors as they occur, enabling the development of explanations about the process [14]. We started from a specific context presentation in order to investigate the phenomenon in depth, in a harmonious configuration between the case study and the grounded theory [1, 16]. As a result of this research, we were able to identify the aspects for the selection of a nurse preceptor.

### Setting

The investigation took place in a hospital in the Lisbon metropolitan area, in the Medicine and the Surgery wards.

### Participants

The participants were 14 nurses from a medicine and a surgery ward. The interviewed nurses were at different stages of the Benner’s professional development model [13] and had different outlooks on nursing preceptorship, in accordance with the diversity required by the chosen methodology.

### Data collection

Data collection took place between November 2018 and October 2019 [1]. The main researcher (PhD student) conducted 14 semi-structured interviews with seven nurses from each ward who agreed to participate after free and informed consent [1].

To collect the data, we developed a script for the interviews. After the pilot test, we made some neccesary adjustments. Together with the head-nurse, we selected nurses with experience in preceptorship and nurses with experience in clinical practice, ensuring there was a wide range of experiences and outlooks concerning the preceptorship. This diversity required further adjustments to the script in order to make the questions relevant for the individuals’ reality. The results of the exploratory interviews guided the preparation of the script for the in-depth interviews.

Before the interview, the nurses previously selected by the head nurses according to the intended diversity were contacted in person to validate their availability to participate in the study.

Before the interview, the nurses were contacted in person to validate their availability to participate in the study. In the first contact, we introduced ourselves, and clarified the information contained in the documents (information to participants and in free and informed consent). We also agreed on the place, date and time of the interview and ensured that the documents (information to participants and free and informed consent) would be sent by e-mail. In the e-mail, we again confirmed the place, date and time of the interview. There were no refusals to participate in the study and nobody dropped out during the study.

The interviews lasted an average of 45 min, were audio recorded and took place at the nurses’ workplaces, with only the interviewee and the researcher present. The interviews were transcribed by the main researcher and emailed to the interviewees for validation.

Convenience sampling was the initial sampling strategy in order to ensure there was an appropriately wide data range. In reaction to the needs identified in data coding, we then used theoretical sampling in order to refine the data results.

Considering that no new data emerged in the last interviews, and after discussion with the second researcher to ensure the reliability and validity of the research, we considered that we had reached theoretical saturation [1, 14]. Therefore, there was no need for follow-up interviews.

The first two exploratory interviews were conducted with nurses considered experts, with experience in both nursing preceptorship and in teaching. After interviewing 8 nurses at different stages of their professional development (beginners, competent, proficient, experts) and with different levels of experience, as well as different outlooks, in preceptorship (no experience, over 10 years of experience, not available for preceptorship), we directed the theoritical sampling process towards selective coding, selecting nurses in different stages of their preceptorship path.

### Data analysis

Data analysis was carried out by the two researchers without the use of software, and began immediately after the first interview. As defined by Strauss and Corbin [14], we started with open coding, and followed with axial and selective coding, always in a logic of reflexivity and constant comparison with theory. In the open coding stage, we used microanalysis (line by line coding), classifying emerging concepts into categories and later into subcategories, which we refined upon in accordance with their properties and dimensions. The categories that emerged were derived from the data. In the axial coding stage, we investigated the relationships between the categories and subcategories, associating and incorporating the data, as well as building explicit and comprehensive explanations. In the selective coding stage, we integrated and refined the categories and subcategories in an interactive dialectic with the data. Throughout this analysis, we built increasingly integrative and complex analytical and conceptual memoranda and diagrams [1, 14].

From the analysis carried out in accordance with the grounded theory methodology [14], the authors identified two concept-generating categories that proved to be fundamental in the selection of the nurse preceptor: skills and individual characteristics.

## Ethical considerations

Research should be held up by ethical principles (namely, beneficence, nonmaleficence, faithfulness, justice, truth-telling, and confidentiality) and it should follow both international and national legislation where it concerns the protection of the fundamental rights of persons.

The research started after receiving permission from the institution and the ethics committee in October 2019 (Minute No. 44/18). The participants were informed of the purpose, benefits and risks before they signed the informed consent form. The participants also verbalized their consent before each interview. Informed consent ensures informed and voluntary participation, safeguarding the participant’s self-determination. It was ensured that this research required no expenses or risk to the participants and that the participants were free to withdraw from the research at any time, with the consequent removal of the interview and the deletion of all data. The anonymity of the participants was guaranteed through the use of identifiers (interview number). Privacy was considered when choosing a location for the interview.

We ensured confidentiality and data security in the codification and in the publishing process, informing the participants that the content of the interview would be digitally archived in a password-protected file, that it would be used solely for this study and that the data would be destroyed within two years of the publication of the research.

We took up the responsibility of securing and protecting the data, ensuring faithfulness and equity in the data processing, as well as information and communication transparency, in view of the participants’ right of confidentiality.

## Rigor/Trustworthiness

The researchers ensured the integrity of the research in three ways: first, by redacting interviewees’ word by word with authenticity and impartiality; second, by being rigorous and honest in coding data and writing results; and third, by respecting the interviewees and the copyright [14, 15].

Data validity, reliability and credibility were assured by resorting to data triangulation and negative case analysis. The credibility of the results also depends on the reproducibility of the research. In this regard, we described in detail and truthfully all the research process and data interpretation, so that it can be reproduced [14, 15].

The description and adequacy of the research process makes it possible to generalize the results, although the explanatory power is more evident in grounded theory. We used memos and diagrams to record the conditions, actions or interactions, and consequences found to support the formulated explanations [14, 15].

During the research process, we first considered the selection of the original sample to ensure diversity. Then, we validated the main categories that emerged and analyzed the indicators that pointed to these categories, while respecting the theoretical formulations to guide the data collection and the theoretical sampling. Afterwards, we reexamined the conceptual relations between categories in order to select the main category [14, 15].

For the empirical grounding of this research, we ensured that the concepts generated in the research were systematically related. We verified the conceptual linkage and density of the categories as well as their development. The theory was built with data variation in mind, including the conditions under which that variation occurs. Throughout the process, we ensured that the data was always appropriately significant [14, 15].

## Results

The results emerged from 14 interviews with seven medicine nurses and seven surgery nurses. In accordance with Benner’s professional development model [12], the interviewees presented a diversity of professional levels. As such, five interviewees were considered experts, three proficient, four competent, one advanced beginner, and one beginner.

In relation to the preceptorship experience, four nurses have been preceptors for over 10 years, two have been preceptors for between five and nine years, four have been preceptors for under four years, two nurses say they are not available to take up the role and two nurses have not yet had the opportunity to take it up. The categories and subcategories identified are shown in Table 1. The categories ‘skills’ and ‘individual characteristics’ emerge as the preceptor’s personal resources, or endogenous resources, which must be enabled in order to mentor the student. Furthermore, the individual characteristics also influence the development of the preceptor’s skills. The key point that keeps resurfacing as an answer to the main question of this study is motivation, in the sense of the drive that leads to the development of both skills and individual characteristics that proved to be fundamental for nursing preceptorship.

**Table 1:** Fundamental categories and subcategories in the selection of nurse preceptor Source: own authorship

Theme	Categories	Sub-categories
Resources	Skills	Communication
		Relational
		Reflective
		Technical-scientific
		Emotional
	Individual characteristics	Perceptiveness
		Responsibility
		Motivation
		Professional initiative

In the skills category, supported by all participants, five subcategories were evidenced:

Communicational skills are fundamental to establish a relationship with the student and to ensure permanent communication that is open and assetive. E8 reinforced these points when they mentioned that *“… the way we communicate, how we get to the other, [how] we lead them to doing it […] I think it’s halfway to success”.*

Relational skills, which are strongly related to communication skills, enable an efficient preceptorship with regular feedback on the student’s performance. E1 referred that being *“[…] able to establish a good relationship with the student…* (E1)” allows *“… a good application of theoretical-practical knowledge […]”* (E1).

Reflective skills are important to analyze the nurse’s practice in order to improve it. E1 valued *“… the ability to analyze, the ability to analyze not only the student but to also my practice […] to analyze myself […]”* and added that this *“… is the main aspect that led me to want to evolve.”*. Reflective skills are also important to analyze the student’s characteristics and style of learning, as well as to assess them. E2 stateed that “… *the assessment that we make of the student […] if I can identify their style of learning […]”* can lead to a *“… better result with that student […]*”.

When it comes to technical-scientific skills, it is recognized that the theoretical knowledge and the professional experience within one’s clinical area are important in preceptorship. E8 mentioned that *“… your professional experience in various areas [makes you] more competent to be able to teach […]*”. E1 added that the nurse “ *[…] must reach a stage where we are experts and then really acquire the status of preceptor.“* and that *“[…] a person who does not clearly master the area of intervention is difficult [sic] to mentor students*.”

Emotional skills help the nurse to begin and maintain a relationship with the student, as well as to manage conflicts and to face challenges. E12 reported that *“… there must be great communication skills here… […]even the issue of empathy and assertiveness….”*.

In the individual characteristics category, also supported by all interviewees, four subcategories emerged:

Perceptiveness, or the ability to observe, is important to integrate attitudes and postures that nurses use in preceptorship and also to observe the performance of the student. E11 stated: *“[…] I started to be more aware of when other colleagues have students, and to take notice of some teachings that they do, you always end up influencing your model of teaching and mentoring a little by imitating colleagues*”.

There must be responsibility not only towards the nurse’s professional practice but also towards the preceptorship, ensuring the safety of the patient, the health team and of the student. E5 underlined that “ *[…] you have to be responsible, you have to be assertive, and you have to be there so that the student also feels safe.”*.

The motivation to be a preceptor, to take on a role one enjoys, gives greater satisfaction to the preceptor, who develops a more efficient relationship with the student and, thus, allows for a more successful preceptorship. E8 values this when he stated *“… that you like to teach, […] that you like to share, to transmit […] pleasure in what you are doing, because that way you learn much better.”.*

Professional initiative, which manifests itself through self-learning and the subsequent professional growth, promotes the development of the nurse’s skills, which are fundamental to the preceptorship. E1 mentioned that *“[…] the expert must really know, have the knowledge, know how to seek knowledge, and know how to apply it, transmit it […]”*.

## Discussion

According to Le Boterf’s [11] concept of skill, nurse preceptors mobilize endogenous and exogenous resources to act effectively and responsibly [13]. Le Boterf [11] mentions that resources can be of a personal nature, and therefore internal to the nurse, or they can be environmental resources, and therefore external to the nurse. The competent management of a preceptorship implies relevant quality action, which is influenced by the nurses’ resources and by the way nurses combine the resources and transpose their skills into action. The categories that emerged (skills and individual characteristics) fit into the personal resources inherent to each preceptor.

## Skills

During the preceptorship, the nurse preceptor has to mobilize communication skills to ensure effective and permanent communication with the student, which promotes the acquisition of knowledge, the ability to solve problems and the development of clinical reasoning [5, 17]. The data found in the study is corroborated by Teferra and Mengistu [18] and Borrallo-Riego et al. [9], who mention that effective communication increases learning, fosters relationships and enables the student to manage emotions. Communication between the nurse preceptor and the student should be assertive, open, permanent, and continuous. The preceptor should consider the student`s learning needs and offer regular feedback on the student`s development 9,19].

Communication is intrinsic to relational skills, and it is fundamental to establish an effective preceptor-student relationship whose objective is to provide supervision and feedback on clinical practice and student performance [9, 18]. The relationship must be effective, strong [20], positive, bidirectional, committed [19], honest and based on responsibility and trust [18, 21]. Lethale et al. [20] and Liu [22] claim that a facilitating and effective relationship can influence the effectiveness of the preceptorship and the learning outcomes by creating a supportive environment, collaboration, trust, encouragement and motivation, which is in agreement with the data we found. Núñez et al. [21], Teferra and Mengistu [18], and Maclaren [23] add that, first, the establishment of strong and effective relationships is valued both by students and preceptors; second, those relationships provide satisfaction to the nurse preceptor; third, it lets the nurse preceptor know that the student trusts them and will seek their support.

Reflective skills imply the preceptor’s capacity for critical thinking in order to analyze and reflect on both his clinical and preceptorship practice. Zuriguel-Perez et al. [24] report that critical thinking is not only one of the basic skills of a nurse and but that it is also related to the knowledge acquired through practice, age, and professional experience. Ferreira et al. [19] corroborate the data found in the aforementioned study when they show that the reflective skills of the preceptor enable three core abilities: first, the ability to reflect on both the professional and preceptorship practices; second, the ability to problematize reality in order to create learning opportunities; third, the ability to analyze and reflect on the student’s characteristics, path, difficulties, needs, development, learning, and assessment. The preceptor’s ability to self-reflect allows the identification of needs, difficulties, limitations and resources to be mobilized to further the process of improving the quality of their preceptorship [25]. Several authors highlight the preceptor’s reflective skills as fundamental for the development of the student`s critical thinking and clinical reasoning [17, 21, 26]. Mikkonen et al. [4] reinforce the importance of the development of these preceptor skills to increase the nursing students’ learning.

Technical-scientific skills integrate theoretical knowledge and clinical practice. Lethale et al. [20] and Ignacio and Chen [5] confirm the data found when they refer that the preceptor, in order to transform clinical experiences into learning experiences, must master clinical practice and the scientific knowledge inherent to it. Ferreira et al. [19] also show that to be productive in teaching, the nurse preceptor has to master clinical practice. Al-Rawajfah et al. [27] add the importance of identifying and appropriately training preceptors to meet the demands of clinical teaching. This professional experience mastery influences the effectiveness of the preceptorship as it enables the mobilization and combination of scientific, technical and teaching skills while conducting the transfer of theoretical knowledge to clinical practice [20]. This study underlines the importance of being an expert nurse [12] at the start of a preceptorship, an aspect supported by Zuriguel-Pérez et al. [24] who mention that professional experience is shown to be related to the development of critical thinking, already mentioned above as fundamental in reflective skills. Teferra and Mengistu [18] also confirm that the preceptor’s knowledge tends to increase with training and preceptorship experience.

Emotional skills emerge as essential for the nurse preceptor’s relationship with both the student and the patients, for the management of conflicts and emotions, and for the response to challenges. The data of this study show that the availability and assertiveness are referred to as essential for the student-preceptor relationship, facilitating learning and the development of skills [9, 21, 28, 29]. The assertiveness inherent to emotional skills presupposes self-confidence, self-esteem, and the absence of communication difficulties. An assertive posture fosters respect, enhances negotiation skills, conflict resolution skills, self-confidence and credibility, thus decreasing student anxiety [4, 8, 28]. Empathy is intrinsically related to altruism and presupposes humane communication as well as active listening [4, 8]. A preceptor’s empathic attitude enables them to understand the student’s behaviors, difficulties, needs and emotions, promoting the reduction of conflicts in the preceptorship [4, 8, 30].

## Individual characteristics

Perceptiveness refers to the nurse’s ability to observe the performance of their peers during the preceptorship, the way they enact it, how they relate to students, how they mobilize clinical reasoning to interrelate information and data. Perceptiveness enables reflection, as well as the assimilation of the role of preceptor and of self-learning processes. The ability to observe is also essential to identify students’ difficulties and needs, and to evaluate their performance [24].

Responsibility must be present in the preceptor, both in relation to their professional practice and during the student preceptorship. The nurse must consider their responsibility towards the patients, in the sense of protecting the human being under their care, and the responsibility towards the health team [21]. A preceptorship also implies the responsibility taken on towards the educational institution and towards the student, within the scope of their learning process [17, 18].

Motivation in preceptorship implies the availability of nurses to assume the role of preceptor and proves to be fundamental to initiate and create a respectful collaborative relationship with the student, a preponderant aspect for the success of the preceptorship [9, 21, 28, 31]. Borrallo-Riego et al. [9] show that the greater the motivation for teaching, the better the students’ learning results. Attitudes and motivation were one of the overarching themes found that reflect the nurses’ professional values [32]. Preceptorship should offer nurses the opportunity to take on a role they like and for which they are apt [8, 21], since, as Núñez et al. [21] point out, vocation emerges as the meaning of being a preceptor. The willingness to teach is also referenced by Drasiku et al. [33] who add that preceptors need support for the teaching role, such as specific professional development and support from educators. Mikkonen et al. [4] add the existence of characteristics that foster motivation and improve the preceptor`s practices, namely empathy, flexibility, tolerance, patience and support.

Professional initiative implies the involvement of nurses in the search and systematization of knowledge and in the developments of skills, aspects already mentioned as essential in preceptorship and student learning [12, 28, 31]. A metasynthesis conducted by Mlambo et al. [32] highlights that nurses value their continuing professional development and consider it to be fundamental to professionalism and lifelong learning. Professional initiative is revealed as being associated with a posture of professional commitment with respect and acceptance of the entire legal and ethical framework that governs the profession and in which the nurse’s responsibility in peer training and preceptorship is evident [2]. Loughran and Koharchik [8] and Núñez et al. [21] show that the professional posture of the nurse, including the way they practise nursing and even their way of living, is transferred to the student.

As previously mentioned, the nurse preceptor is a facilitator in the learning and the development of nursing students’ skills, and, to this end, it is essential to act competently for the success of the preceptorship.

Therefore, the nurses identified a set of personal resources that they could mobilize and transfer to act effectively as nurse preceptors, and that should be taken into account when choosing the nurse preceptor.

## Conclusion

In nursing preceptorship, nurses value as assets not only the individual characteristics and skills they have, but also the ones they developed throughout their personal, academic, and professional career.

The selection of the nurse preceptor could consider a set of aspects that emerged from this study, namely, skills (communication skills, relational skills, reflexive skills, technical-scientific skills, and emotional skills) and individual characteristics (perceptiveness, responsibility, motivation, and professional initiative).

The identification and recognition of these aspects for the selection of nurse preceptors allows reflection and informed decision-making, with a view to the success of the preceptorship process as well as to the increase of the quality of teaching and of the nursing students’ learning. These aspects can also contribute to the development of nurse preceptor’s training programs.

The fact the research is set as a case study format in two specific contexts (medicine and surgery) presents itself as a limitation since the results can only be generalised from an analytical point of view.

## Data Availability

The datasets used and/or analyzed during the current study are available from the corresponding author on reasonable request.
